# Detection and Phylogenetic Analyses of Taura Syndrome Virus from Archived Davidson’s-Fixed Paraffin-Embedded Shrimp Tissue

**DOI:** 10.3390/v12091030

**Published:** 2020-09-16

**Authors:** Lauren Marie Ochoa, Roberto Cruz-Flores, Arun K. Dhar

**Affiliations:** Aquaculture Pathology Laboratory, School of Animal and Comparative Biomedical Sciences, University of Arizona, Tucson, AZ 85721, USA; lauren33@email.arizona.edu (L.M.O.); robertocruz@arizona.edu (R.C.-F.)

**Keywords:** Taura syndrome virus, aquaculture, shrimp

## Abstract

Taura syndrome is a World Organization for Animal Health (OIE)-listed disease of marine shrimp that is caused by Taura syndrome virus (TSV), a single-stranded RNA virus. Here we demonstrate the utility of using 15-year-old archived Davidson’s-fixed paraffin-embedded (DFPE) shrimp tissues for TSV detection and phylogenetic analyses. Total RNA was isolated from known TSV-infected DFPE tissues using three commercially available kits and the purity and ability to detect TSV in the isolated RNA were compared. TSV was successfully detected through RT-qPCR in all the tested samples. Among the TSV-specific primers screened through RT-PCR, primer pair TSV-20 for the RNA-dependent RNA polymerase (RdRp), primers TSV-15 and TSV-16 for the capsid protein gene VP2 and primers TSV-5 for the capsid protein gene VP1 amplified the highest number of samples. To assess the phylogenetic relation among different TSV isolates, the VP1 gene was amplified and sequenced in overlapping segments. Concatenated sequences from smaller fragments were taken for phylogenetic analyses. The results showed that the TSV isolates from this study generally clustered with homologous isolates from the corresponding geographical regions indicating RNA derived from DFPE tissues can be used for pathogen detection and retrospective analyses. The ability to perform genomic characterization from archived tissue will expedite pathogen discovery, development of diagnostic tools and prevent disease spread in shrimp and potentially other aquaculture species worldwide.

## 1. Introduction

Taura syndrome (TS), caused by Taura syndrome virus (TSV) is a disease of penaeid shrimp that is estimated to have caused between USD 1.2–2 billion worth of economic losses in the Americas during its widespread panzootic between 1992 and 1996 [1,2,3]. Taura syndrome is a World Organization for Animal Health (OIE)-listed disease that has been detected on five continents [4]. This pathogen is a non-enveloped single-stranded RNA (~10 kb genome) containing a virus classified as a member of the family *Dicistroviridae*, genus *Aparavirus* [5]. The detection of shrimp pathogens such as TSV is critical for controlling, predicting and preventing potential outbreaks and large economic losses.

In studying shrimp diseases, Davidson’s-fixed paraffin-embedded (DFPE) tissues are considered prized biological samples for histological analysis. These samples are often the only tissues available for retrospective studies, yet no work has been done with the detection of RNA viral pathogens from archived DFPE shrimp tissues. Despite the ease and convenience of DFPE tissue storage, the recovery of nucleic acids from shrimp tissue specimens is particularly challenging. The fixative process leads to the cross-linking between DNA/RNA and proteins and contributes to the fragmentation of both DNA and RNA [6]. When working with archival DFPE shrimp tissue, there is an additional challenge because shrimp tissues are fixed in Davidson’s fixative instead of formalin. Davidson’s fixative contains acetic acid to soften the shrimp exoskeleton, which further contributes to nucleic acid degradation through acid hydrolysis. These changes interfere with many classical molecular analyses requiring high quality nucleic acids. The negative effects of Davidson’s fixative on virus detection by in situ hybridization using non-radiolabeled probes have been discussed while detecting TSV [7] and infectious myonecrosis virus (IMNV) in shrimp [8]. These studies highlight the challenges when working with DFPE shrimp tissues.

Formalin-fixed paraffin-embedded (FFPE) tissues are similar to DFPE tissues but are fixed in formalin instead of Davidson’s fixative. Although there are challenges when working with both FFPE and DFPE tissues, researchers have recently extracted nucleic acids from FFPE tissues for pathogen detection while studying human and animal diseases. For example, researchers utilized RNA isolated from FFPE tissues and next generation sequencing (NGS) to discover a novel strain of rotavirus [6]. FFPE tissues were used to reconstruct the 1918 Spanish influenza virus genome and compare the viral sequences with a recent H1N1 strain to identify the unique high-virulence phenotype observed with the pandemic virus [9]. In human cancer studies, a large collection of diseased and normal tissue stored at hospitals has provided an excellent source for molecular genetic studies [10]. In shrimp disease research, Cruz-Flores et al. [11] have recently reconstructed the genome of white spot syndrome virus (WSSV), a dsDNA containing virus from DFPE shrimp tissue confirming the viability of using these types of tissues for retrospective studies and pathogen characterization.

In the present study, the utility of DFPE tissue for an RNA viral pathogen detection was evaluated using TSV as a proof-of-concept (POC) study. Twenty-nine known TSV-infected DFPE tissues archived for 15 years and representing different geographical locations were utilized to isolate total RNA. Initially, TSV was successfully detected through real-time RT-PCR. Subsequently, a series of primers designed to target the non-structural and structural genes provided successful detection of TSV by conventional RT-PCR. Phylogenetic analyses using capsid protein gene VP1 sequences showed the genetic relatedness of TSV isolates from different geographical regions with corresponding isolates for which sequences were available in GenBank. The data presented here further established the utility of DFPE tissues as a biological resource for pathogen detection and evolutionary studies. To our knowledge, this is the first report of the use of using DFPE tissue to detect and conduct genetic analyses of RNA viruses infecting shrimp and opens a new avenue for pathogen detection and characterization in shrimp aquaculture and potentially in other aquatic species.

## 2. Materials and Methods

### 2.1. Sample Selection

**Figure 1:**
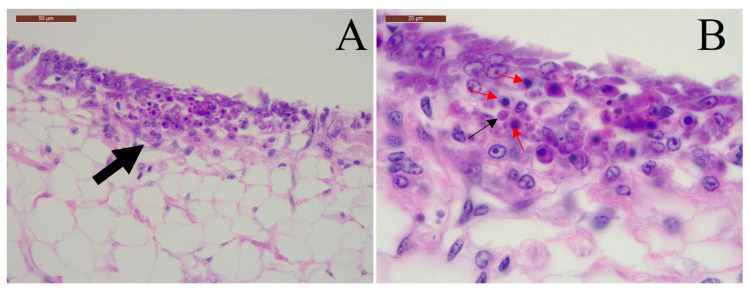
Taura syndrome virus (TSV) infection in *Litopenaeus vannamei*. (**A**) Focal acute-phase infection (large arrow) in the cuticular epithelium characterized by spherical intracytoplasmic inclusions bodies, pyknosis and karyorrhexis. Scale bar, 50 μM. (**B**) High magnification of the same section; the basophilic intracytoplasmic inclusions bodies (black arrow) and pyknotic nuclei (red arrow) are shown. Scale bar, 20 μM.

A histological analysis of archived DFPE tissue blocks of known TSV cases in the Aquaculture Pathology Laboratory from 2005 was performed. The samples that presented TSV infections with a severity level of Grades 3 to 4 were selected for total RNA extraction (*n* = 29) due to the potentially higher amount of viral nucleic acids present (Figure 1). The severity of the TSV infection was graded based on a semi-quantitative scale that ranged from Grade 0 to Grade 4 [12]. Grade 3 TSV infection showed moderate to high signs of disease shown by the number and severity of the pathogen caused lesions and Grade 4 TSV infection showed high numbers of pathogen caused lesions and tissue destruction. A microtome was used to cut three sections from archived DFPE tissue 20 µm in thickness from each sample. Three consecutive sections of 20 µm in thickness were used for molecular analyses and efforts were made to ensure that an equivalent amount of tissue was taken from each paraffin block. For the purpose of this study, ‘case’ and ‘sample’ have been used interchangeably. A summary of samples taken for RNA isolation and RT-PCR amplification is shown in Table 1.

### 2.2. RNA Extraction and Quality Assessment

Total RNA extraction was performed using three commercially available kits: Invitrogen PureLink FFPE RNA Isolation Kit (Invitrogen, Carlsbad, CA, USA), Norgen Biotek FFPE RNA Purification Kit (Norgen Biotek, Thorold, ON, Canada) and Qiagen RNeasy FFPE Kit (Qiagen, Hilden, Germany), in accordance with the manufacturer’s recommendations. To assess total RNA quantity and quality, 1 µL of RNA was utilized in triplicate to obtain a mean value for RNA concentration, 260/280 and 260/230 ratios using a NanoDrop™ 2000 (Thermo Fisher Scientific, Waltham, MA, USA). All reactions were performed in a sterile environment and the isolated RNA was stored at −20 °C until further use. In addition, three representative RNA samples extracted with the Qiagen RNeasy FFPE Kit were analyzed using an automated electrophoresis TapeStation system (Agilent, Santa Clara, CA, USA) to investigate the degree of RNA degradation (Supplementary Materials Figure S1).

### 2.3. Detection of TSV through Real-Time RT-PCR (RT-qPCR)

RNA samples were heated to 90 ± 2 °C then immediately quenched. Real-time reverse transcription-polymerase chain reactions (RT-qPCR) were performed following a protocol recommended by The World Organization for Animal Health (OIE, Paris, France) for the detection of TSV [13]. A reaction mixture was prepared consisting of 2.5 µL of TaqMan™ Fast Virus 1-Step Master Mix (Applied Biosystem, Foster City, CA, USA), 1 µL of forward and reverse primers (5 μM each), 0.5 µL of TaqMan probe (2 μM), 2 µL of RNA and 4 µL of HPLC grade water in a final reaction volume of 10 µL. TSV primers 1004F (5′-TTG GGC ACC CGA CAT T-3′) and 1075R (5′-GGG AGC TTA AACTGG ACA CAC TGT-3′) and a TSV probe (FAM-CAG CAC TGA CGC ACA ATA TTC GAG CAT C-TAMARA) were used to amplify a 72 bp amplicon (Table 2). RT-qPCR was performed with an Applied Biosystems Step One Plus^®^ real-time PCR machine. Thermocycling conditions consisted of a reverse transcription step at 48 °C for 5 min and a denaturation step at 95 °C for 20 s followed by an amplification step at 40 cycles of 95 °C for 1 s and 60 °C for 20 s. Each sample was run in triplicate and a TSV-positive control, a negative control representing RNA isolated from a specific pathogen free (SPF) shrimp and a no template control were used for all RT-qPCR detections.

### 2.4. Statistical Analysis

Statistical analysis of the mean Ct (cycle threshold) values was performed using the Kruskal–Wallis non-parametric test with SPSS v16.0 software.

### 2.5. Detection of Eukaryotic Translation Elongation Factor 1 Alpha (EF-1α) through Real-Time RT-PCR

The elongation factor 1-α (EF-1α) gene from shrimp was selected as an internal control gene [14] to determine if inhibitory substances were present in RNA isolated from DFPE tissue. The mRNA expression of EF-1α was measured by quantitative RT-qPCR using PowerUp™ SYBR^®^ Green Master Mix (Applied Biosystems). The cDNA synthesis was performed using a Tetro cDNA Synthesis Kit (Bioline Meridian Bioscience, London, UK) following the manufacturer’s protocol. Briefly, 2 µL of total RNA from each sample (*n* = 29) was combined with a reaction mixture comprised of 1 µL random hexamer, 4 µL 10 µM dNTP, 1 µL 5× RT Buffer, 1 µL RiboSafe RNase Inhibitor, 1 µL Tetro Reverse Transcriptase and 12 µL DEPC-treated water in a final reaction volume of 20 µL. The cDNA was diluted to 50 ng/ µL. Then RT-qPCR was performed with an Applied Biosystems Step One Plus^®^ real-time PCR machine. The EF-1α primers 123F (5′-TCGCCGAACTGCTGACCAAGA-3′) and 123R (5′-CCGGCTTCCAGTTCCTTACC-3′) were used to generate a 55 bp amplicon as described by Dhar et al. [14]. The RT-qPCR mixture contained 7 µL of PowerUp™ SYBR^®^ Green Master Mix, 1 µL each of forward and reverse primers (10 µM) and 1 µL of cDNA. The Applied Biosystems Step One Plus^®^ program consisted of an initial denaturation step at 95 °C for 3 min followed by 40 cycles of denaturation at 95 °C for 10 s, annealing for 30 s at 55 °C and extension for 30 s at 72 °C. Following amplification, the melt curve analysis was performed. The reaction temperature was increased to 95 °C for 15s then decreased to 60 °C for 1 min and increased to 95 °C at a rate 0.3 °C per second with a continuous fluorescence monitoring. Each sample was run in triplicate in a 96 well plate and the mean Ct value was used for further analysis.

### 2.6. TSV Primer Design

TSV-specific primers for conventional RT-PCR were designed using Geneious Prime 2019.2.1 (Biomatters, Auckland, New Zealand) and the TSV reference genome (GenBank accession: NC_003005) (Hawaii, USA). Each set of forward and reverse primers was designed to amplify between 100–150 bp overlapping genome segments. Fourteen sets of primers were designed to amplify the VP1 Hawaiigene in Open Reading Frame (ORF) 2 from 7901–9176 nt to use for phylogenetic analysis. To amplify more conserved regions of the TSV genome, three sets of primers were designed to amplify a portion of the VP2 gene in ORF2 from 6948–7252 nt and four sets of primers were also designed to amplify portions of RpRd gene in ORF1 from 5194–6610 nt. A summary of these primers is presented in Table 2. Each sample was screened with all the designed primers including the OIE-recommended primers.

### 2.7. Amplification of TSV by RT-PCR and Amplicon Sequencing

Total RNA isolated from Norgen Biotek FFPE RNA Purification Kit and Qiagen RNeasy FFPE Kit were selected to carry out RT-PCR due to the superior performance of these two kits in isolating high quality RNA (see Result section below). Complementary DNA (cDNA) was synthesized and utilized for RT-PCR amplification of TSV VP1, VP2 and RdRp genes. A total of 1218 samples were tested for TSV detection via RT-PCR for this study. Amplicons were generated by 21 sets of primers used to amplify 29 samples each from the Norgen Biotek FFPE RNA Purification Kit and the Qiagen RNeasy FFPE Kit (21 primers × 29 samples × 2 extraction kits = 1218 total amplicons). For PCR amplification, 1 µL of each cDNA sample was combined with 1 µL forward and reverse primers (5 μM each) and 12.5 µL of DreamTaq™ Hot Start Green PCR Master Mix (Thermo Fisher Scientific) in a 25 µL reaction volume. Thermocycling conditions consisted of 95 °C for 5 min followed by 35 cycles of 95 °C for 30 s, 60 °C for 30 s and 72 °C for 30 s then 72 °C for 5 min. A TSV-positive control, a negative control representing RNA isolated from a specific pathogen free (SPF) shrimp and a no template control were used for all PCR amplifications. A housekeeping gene, *EF-1α*, was used as an internal control. The amplicons were electrophoresed in a 1.5% agarose gel for 60 min at 80 V and were visualized on a GelDoc™ XR+ (Bio-Rad, Hercules, CA, USA). PCR amplicons were sequenced using the Sanger sequencing method (University of Arizona Genetics Core, Tucson, AZ, USA) and Geneious Prime 2019.2.1 was used to trim and align the sequences to the TSV reference genome (NC_003005). Nucleotide sequence identity was determined using the BLASTn of NCBI (BLAST+2.10.1).

### 2.8. Phylogenetic Analysis

As not every case yielded the same amplification results, only samples that amplified the selected four primer pairs (TSV1, TSV12, TSV 13 and TSV 14) were chosen for phylogenetic analysis. Twelve (4, 5, 6, 8, 10, 14, 15, 16, 17, 18, 21 and 27; see Table 1) out of 29 representative samples from different geographical regions were used to generate concatenated consensus sequences of the VP1 gene. The concatenated consensus sequences of the VP1 gene (150 nt) were compared with the homologous concatenated gene sequence of 29 TSV isolates from varying geographical locations that are available in GenBank. Isolates selected from GenBank are summarized in Table 3. A multiple alignment was performed using the Geneious Prime program and the CLUSTALW Plugin using the 12 samples from this study and the 29 isolates acquired from GenBank. Phylogenetic analysis was conducted using MEGA 10.0.5. A neighbor-joining phylogenetic tree [15] was constructed using the multiple alignment and a bootstrap consensus was inferred from 1000 replicates.

## 3. Results

### 3.1. RNA Quantity and Quality Assessment

**Figure 2:**
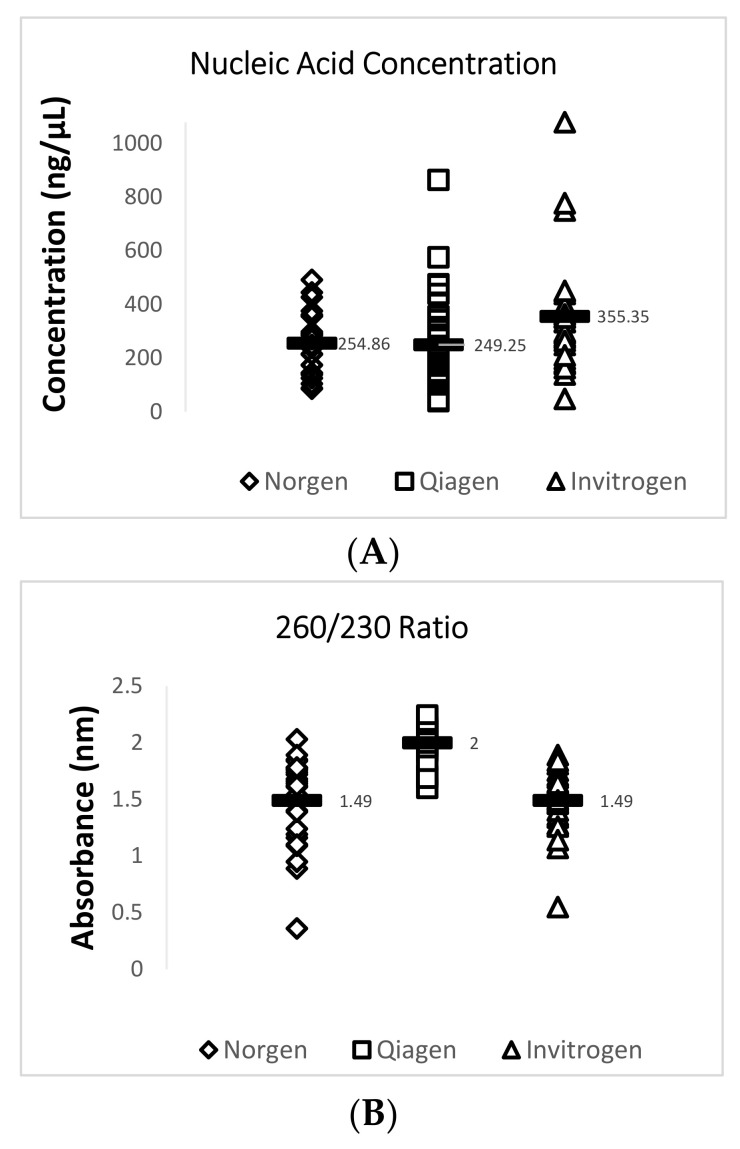

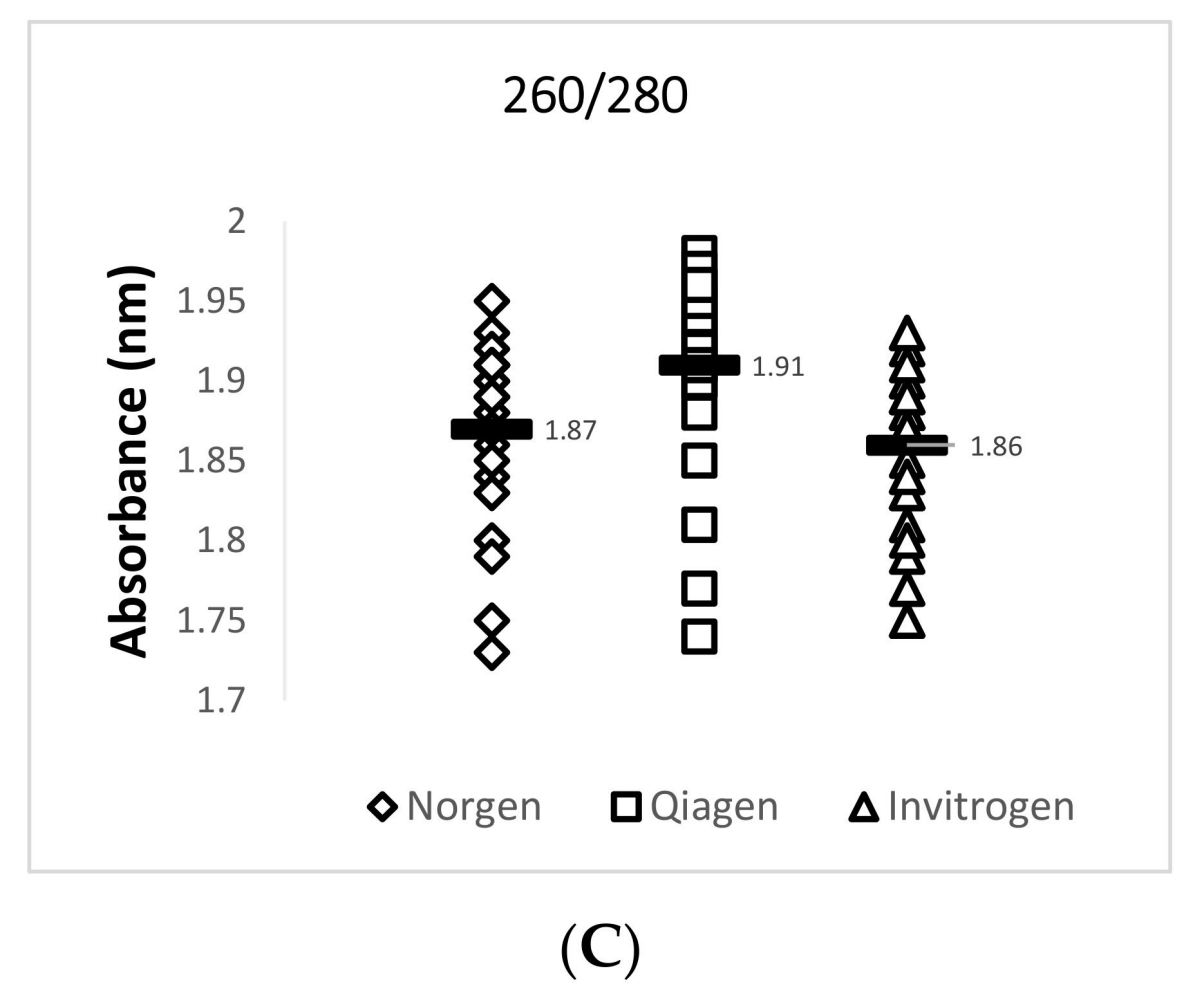
Quantity and quality assessment of total RNA isolated from archived TSV-infected Davidson’s-fixed paraffin-embedded (DFPE) shrimp tissues. Each test was run in triplicate and a mean value for each data set was obtained. The figure in (**A**) shows a comparison of the mean nucleic acid concentrations for 29 samples from each extraction kit. The figure in (**B**) shows a comparison of the mean 260/230 ratio values for 29 samples from each extraction kit. The figure in (**C**) shows a comparison of the mean 260/280 ratio values for the 29 samples from each extraction kit. The horizontal line in each group represents a geometric mean of the data in the corresponding vertical data set.

Data obtained from the NanoDrop™ 2000 show that the Invitrogen PureLink FFPE RNA Isolation Kit yielded the highest mean concentration value of 355.3 ng/µL for total RNA while the Qiagen RNeasy FFPE Kit yielded the lowest mean concentration value of 249.2 ng/µL. The Qiagen RNeasy FFPE Kit yielded the best mean 260/280 (1.91) and 260/230 (2.00) ratio values when compared with the two other extraction kits. A 260/280 ratio value of 2.0 is generally accepted as “pure” for RNA and a 260/230 ratio value of 2.0–2.2 is generally accepted as “pure” for nucleic acids [16]. Lower ratio values may indicate a higher presence of contaminants such as phenols or proteins. A summary of this data is provided in Figure 2 and Table 4.

### 3.2. Detection of TSV Using RT-qPCR

**Figure 3:**
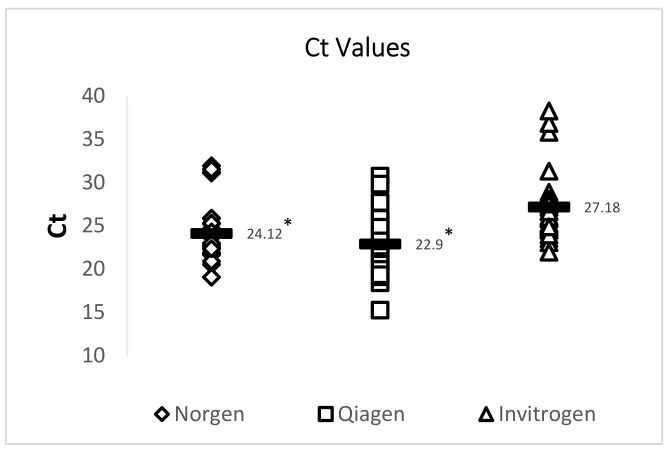
Analysis of the Ct values of TSV amplicons generated by RT-qPCR. Three commercially available extraction kits were utilized for total RNA extraction and total RNA isolated from archived TSV-infected DFPE shrimp tissues was used to generate a 72 bp amplicon of the TSV genome. The horizontal line in each group represents a geometric mean of the Ct values of the corresponding vertical data set. Asterisks represent statistical significance (*p* < 0.05).

A 72 bp cDNA amplicon in TSV ORF1 was amplified by TaqMan RT-qPCR following an OIE-recommended protocol. RT-qPCR results showed that both the Qiagen RNeasy FFPE Kit and the Norgen Biotek FFPE RNA Purification Kit yielded RNA that provided significantly lower Ct values than RNA isolated using an Invitrogen PureLink FFPE RNA Isolation Kit. While all 29 samples were successfully amplified using RNA isolated by using the Qiagen RNeasy FFPE Kit and the Norgen Biotek FFPE RNA Purification Kit, two out of the 29 samples did not provide TSV amplification when RNA isolated using the Invitrogen PureLink FFPE RNA Isolation Kit was used. A summary of this data is provided in Figure 3 and Table 4.

### 3.3. Detection of TSV Capsid Protein Genes (VP1 and VP2), RdRp and Internal Control Gene EF-1α by Conventional RT-PCR

**Figure 4:**
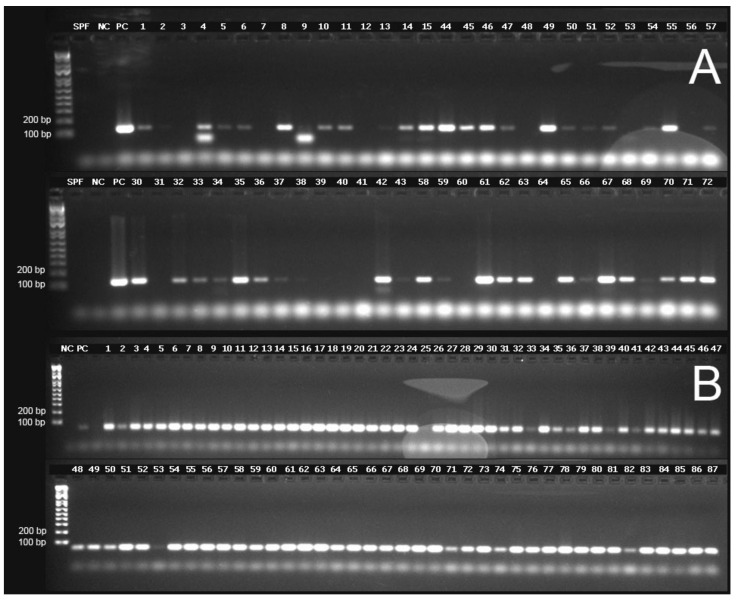
Representative agarose gel electrophoresis images for the detection of TSV genes and the internal control gene *EF-1α* (elongation factor 1-α). (**A**) TSV VP1 gene (primer pairs TSV-5, amplicon size 122 bp). (**B**) Shrimp *EF-1α* gene. SPF = Specific Pathogen Free negative control, NC = no template control, PC = TSV positive control. The first lane in each gel represents a molecular weight marker (Invitrogen 1 kb Plus DNA Ladder).

The TSV capsid protein genes VP1 and VP2, the TSV RdRp gene and the shrimp internal control gene EF-1α were successfully amplified by conventional RT-PCR using RNA isolated from DFPE tissue and following the Qiagen RNeasy FFPE Kit protocol. However, the number of samples that amplified varied depending on the target gene and primers used to amplify the corresponding genes. A summary of the amplification of the TSV genes using the different primer sets is presented in Supplementary Table S1. The internal control gene *EF-1α* was successfully amplified in all but one case (Sample No. 25), which could be due to low concentration (43.4 ng/µL), low 260/280 (1.74) and low 260/230 ratios (1.51) or the presence of any PCR inhibitor in this sample. Subsequently, this sample (Sample No. 25) was excluded from the RT-PCR data analysis. A representative gel image of the amplified segments of TSV VP1 and the internal control gene, *EF-1α*, can be seen in Figure 4. It is worth noting that some samples (Samples 4 and 9) had smaller band size than the expected amplicon (Figure 4). Since the smaller sized amplicons were not sequenced, the origin of these bands cannot be determined.

**Figure 5:**
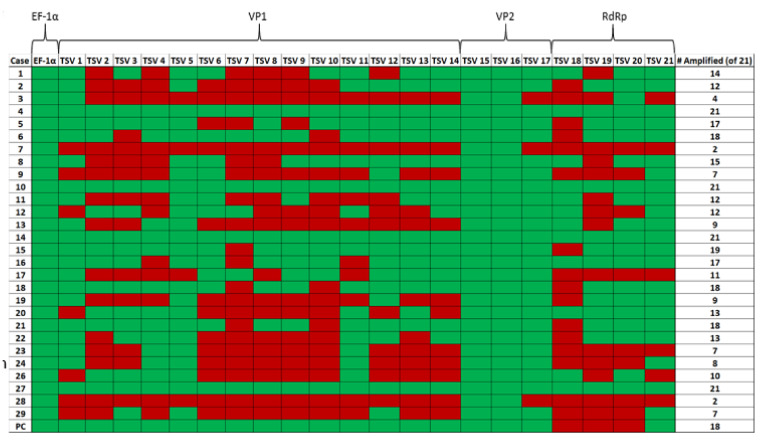
A pictograph of the RT-PCR amplification of TSV capsid protein VP1 and VP2 genes, RNA-dependent RNA polymerase (RdRp) and shrimp internal control gene *EF-1α*. The primer pairs (TSV-1 to TSV-21) used to amplify different TSV genes are shown on the top row of the map. The case numbers are indicated in the far-left column (excluding Sample No. 25, as mentioned previously) and the total number of primer pairs (out of 21) that were successful in amplifying each sample can be seen in the far-right column. Green boxes indicate amplification and red boxes indicate no amplification for the corresponding primers.

Samples 4, 10, 14 and 27 (Figure 5) provided successful amplification of all selected regions of the genome. Among the three TSV genes targeted for amplification, the VP2 gene in ORF2 was the most successfully amplified gene among the samples tested (28 out of 28 samples amplified by using primer pairs TSV-15 and TSV-16). This was followed by the TSV VP1 gene (25 out of 28 samples amplified using the primer set TSV-5) and RdRp gene (20 out of 28 samples amplified using the primer set TSV-20). Finally, only four out of 28 samples were successfully amplified with the OIE-recommended primers (Supplementary Materials Table S1). Figure 5 is a pictograph that represents the entire amplification data obtained from the RT-PCR amplification of the different segments of the TSV genome. The RT-PCR data revealed that successful amplification occurred when the amplicon size ranged between 100 and 150 bp and when the amplicon size was increased over 150 bp, the efficiency of the amplification was reduced.

A summary of sample numbers and their corresponding amplification results is presented in Table 5. Four out of 28 samples (14.3%, sample numbers 4, 10, 14 and 27) provided amplification of all 21 amplicons. Six samples (21.4%, sample numbers 5, 6, 15, 16, 18 and 21) provided amplification of 17 to 19 out of 21 amplicons. Seven samples (25%, sample numbers 1, 2, 8, 11, 12, 20 and 22) provided amplification of 12–16 out of 21 amplicons. The remaining 11 samples (39.3%) provided anywhere between 2 to 11 out of a total of 21 amplicons.

### 3.4. Phylogenetic Analysis

**Figure 6:**
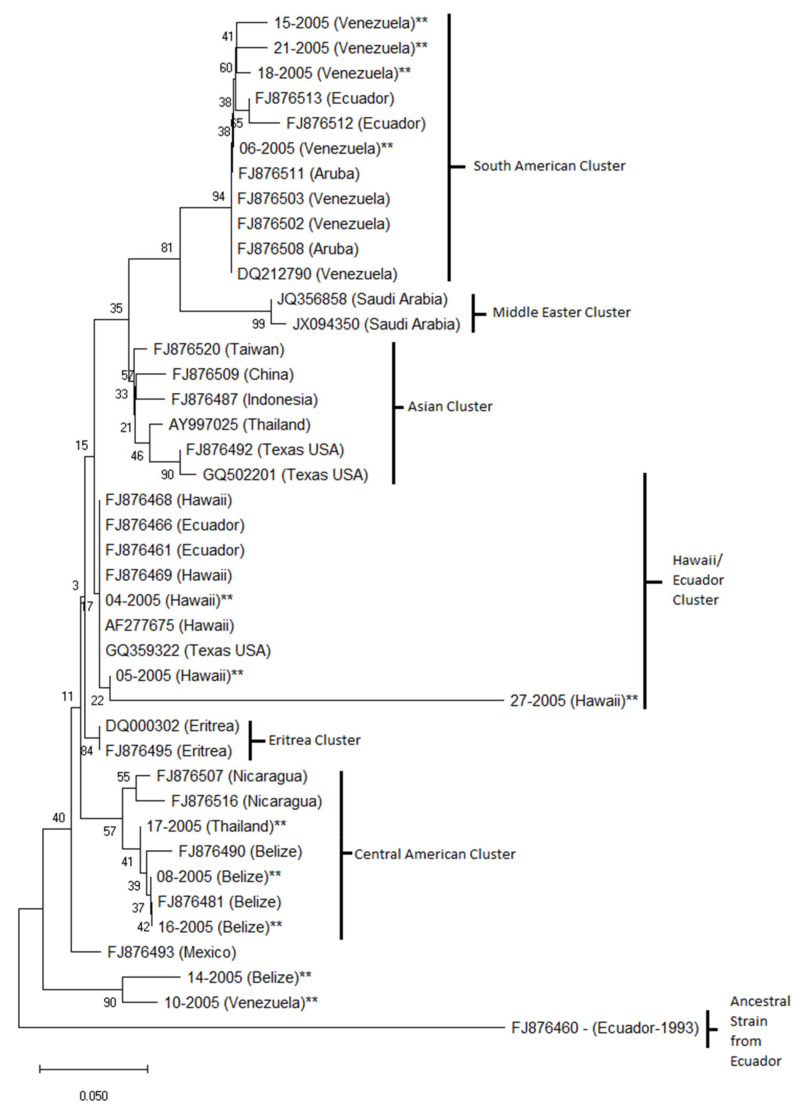
Phylogenetic analysis of TSV isolates. The evolutionary history was inferred using the neighbor-joining method. The optimal tree with the sum of branch length = 0.87629497 is shown. The percentage of replicate trees in which the associated taxa clustered together in the bootstrap test (1000 replicates) are shown next to the branches. The tree is drawn to scale with branch lengths in the same units as those of the evolutionary distances used to infer the phylogenetic tree. The evolutionary distances were computed using the Maximum Composite Likelihood method and are in the units of the number of base substitutions per site. This analysis included 12 samples from the present study (denoted by ** on the tree) and 29 GenBank accessions.

A neighbor-joining phylogenetic tree constructed using the VP1 nucleotide sequence showed that the Venezuela isolates 06-2005, 15-2005, 18-2005 and 21-2005 formed a well-supported South American cluster with GenBank isolates from Venezuela, Aruba and Ecuador (Figure 6). TSV isolates from Taiwan, China, Indonesia, Thailand and Texas, USA, for which sequences are available in Genbank, formed the Asian cluster. Hawaiian isolates 04-2005, 05-2005 and 27-2005 from the present study clustered with GenBank isolates from Hawaii and Ecuador to form the Hawaii/Ecuador cluster. Eritrea isolates formed a separate well-supported Eritrea cluster. Thailand isolate 17-2005 clustered with GenBank isolates from Nicaragua and Belize forming the Central American cluster. Interestingly, isolates 14-2005 and 10-2005 formed a separate, relatively well-supported cluster. The ancestral GenBank isolate from Ecuador from 1993 formed an independent cluster. The alignment used to generate the phylogenetic tree is provided as a supplementary file.

## 4. Discussion

In the aquaculture industry, the detection of infectious diseases in a timely manner is critical for predicting, preventing and controlling potential outbreaks and large economic losses. DFPE tissues are often the only samples available for retrospective studies for determining the origin, evolution and spread of pathogens across countries and continents. While DFPE tissues can be extremely valuable in addressing these questions, retrieving genetic information from DFPE histological samples has presented significant challenges in pathogen discovery and genetic studies in humans and animals. This is the first study that demonstrates the utility of using archived DFPE histological blocks for shrimp RNA viral pathogen detection, using TSV as a model.

Three different commercially available kits were used to isolate total RNA from DFPE shrimp tissue to detect TSV. Although the Invitrogen PureLink FFPE RNA Isolation Kit yielded the highest mean concentration, the Qiagen RNeasy FFPE Kit provided RNA that yielded the lowest mean Ct value while detecting TSV by TaqMan RT-qPCR. Total RNA isolated using a Qiagen RNeasy FFPE Kit had the best 260/280 and 260/230 values of 1.91 and 2.0, respectively. These data indicate that the RNA obtained with the Qiagen RNeasy FFPE Kit was purer than the RNA obtained with the other FFPE RNA isolation kits. However, we should point out that further studies need to be performed using a non-photometric approach (Qubit quantifier) that could indicate if potential DNA contamination is present.

Total RNA isolated from DFPE shrimp tissue was subjected to the detection of TSV by RT-qPCR following an OIE-recommended method and conventional RT-PCR. The amplicon size for TaqMan RT-qPCR was 72 bp and the size range of the amplicons in conventional RT-PCR was between 100–231 bp. While results from RT-qPCR showed that all 29 samples tested TSV positive, conventional RT-PCR data were more variable among the samples tested. The results showed that the smaller amplicons (100–150 bp) were most successfully detected through RT-PCR and amplicon sizes greater than 150 bp, including a 231 bp amplicon that was amplified using an OIE-recommended protocol [17], could not be amplified from all samples. This is most likely due to chemical modification, cross-linking of RNA and proteins, and RNA fragmentation that is seen when extracting nucleic acids from DFPE tissues. The three representative samples analyzed using automated electrophoresis (TapeStation system, Agilent) gave RNA Integrity Numbers (RIN) that ranged from 1.7–2.6, indicating a high level of fragmentation. While amplifying the VP1 gene, we noticed some samples (Samples 4 and 9, see Figure 4) provided a smaller amplicon in addition to the expected size amplicon. It is possible that these amplicons represented non-specific amplifications. Alternatively, the smaller sized amplicon may have represented a deletion in the PCR target region in the VP1 gene. Since both samples also provided an expected size amplicon, albeit faint bands, it is also possible that the two amplicons represented a wild type and a mutated genotype of TSV.

The RT-PCR results showed that amplicons originating from the VP2 gene in the TSV genome provided the most successful amplification while amplicons originating from the VP1 gene were the least successful. This is likely because the VP2 gene is a more conserved region than the VP1 gene region [4]. According to Wertheim et al. [4], the TSV nucleotide substitution rate in the VP1 region was approximately 2.37 × 10^−3^ substitution/site/year. It is also interesting to note that primers designed to amplify the RdRp region of the TSV genome were not as successful as the VP2 gene region in terms of total percent of amplified samples. This could be due to fragmentation or chemical modification in the RdRp region during the fixative process or to the mutations in this region where the TSV-specific primers were intended to bind. Based on the overall amplification success in 28 DFPE tissue samples detected via RT-PCR, it can be concluded that primers designed based on the VP2 gene with an amplicon size of less than 150 bp were optimal for detecting TSV from archived DFPE tissues.

The VP1 gene was selected for phylogenetic analysis because it is a highly variable region of the genome [18]. The phylogenetic analysis presented here showed a similar topology to previously published TSV phylogenetic studies. For example, a South American cluster, consisting of isolates from Venezuela, Ecuador and Aruba, was consistent with the structure shown by Aranguren et al. [19]. The Hawaii/Ecuador cluster formed by isolates from Hawaii and Ecuador was observed in the present study and in the study by Aranguren et al. [19]. A Central American and Asian cluster could also be observed. Similarly, GenBank isolates from Texas, USA clustered with the Asian isolates, which was consistent with the structure observed by Dhar et al. [20]. A separate Middle Eastern cluster, consisting of two Saudi Arabian isolates, was similar to the structure shown by Tang et al. [21].

It is important to note that two of the isolates in this study were observed in different clusters than what was expected (17-2005 from Thailand and 10-2005 from Venezuela). Dhar et al. [20] carried out the genetic characterization of a TSV isolate affecting a cultured shrimp population in Texas. The Texas isolates were more closely related to the isolates from China and Thailand than to the Hawaii isolate. It is well known that the movement of infected shrimp is the main cause for the spread of disease in shrimp aquaculture worldwide [2]. We hypothesize that while the 17-2005 isolate from Thailand lies within the Central American cluster and the 10-2005 isolate from Venezuela forms a sub-cluster within the Central American cluster with isolate 14-2005 from Belize, these TSV isolates could have been introduced from a distinct geographical location such as Thailand or Venezuela.

It is fundamental to point out that for the phylogenetic analysis, the concatenated consensus sequences were 150 nt in length. It is well known that sequences less than 800 nt in length are less informative for phylogenetic analysis due to the limited information that these shorter sequences can yield [22]. However, in our study, even though sequence length was not optimal, the overall topology of the phylogenetic tree showed a resemblance to previous studies. To obtain higher support for the distinct clusters, new primers could be designed to amplify and sequence the regions not used in this study and the new data set could be used for further refining the phylogenetic analysis.

The results from the present study demonstrate that both health assessment and targeted pathogen screening in shrimp can be done using DFPE tissue samples that are routinely used for histological analyses. The feasibility to detect viral pathogens from archived DFPE blocks opens up unlimited possibilities for retrospective studies and the discovery of novel pathogens. These applications have direct implications in disease management in shrimp aquaculture and the potential to be applied to pathogens that affect other aquaculture species.

## Figures and Tables

**Table 1:** Archived Davidson’s-fixed paraffin-embedded (DFPE) tissue samples selected for analyses. The cases (*n* = 29) are displayed with their corresponding sample numbers from each of the three extraction kits (Invitrogen PureLink FFPE RNA Isolation Kit, Norgen Biotek FFPE RNA Purification Kit and Qiagen RNeasy FFPE Kit), the place of origin, the year each case was received, shrimp species and grade of infection (from G1 to G4, where G4 represents the highest level of TSV infection.

Case No.	Norgen KitSample #	Qiagen KitSample #	Invitrogen KitSample #	Year	Origin	Species	Grade of Infection
1	1	58	73	2005	Belize	*L. vannamei*	G3
2	2	59	74	2005	Venezuela	*L. vannamei*	G3, G4
3	3	60	75	2005	Thailand	*P. monodon*	G3, G4
4	4	61	76	2005	Hawaii	*L. vannamei*	G2–4
5	5	62	77	2005	Hawaii	*L. vannamei*	G3, G4
6	6	63	78	2005	Venezuela	*L. vannamei*	G3
7	7	64	79	2005	Thailand	*P. monodon*	G3
8	8	65	80	2005	Belize	*L. vannamei*	G3
9	9	66	81	2005	Hawaii	*L. vannamei*	G3, G4
10	10	67	82	2005	Venezuela	*L. vannamei*	G3, G4
11	11	68	83	2005	Belize	*L. vannamei*	G4
12	12	69	84	2005	Thailand	*L. vannamei*	G4
13	13	70	85	2005	Belize	*L. vannamei*	G4
14	14	71	86	2005	Belize	*L. vannamei*	G3, G4
15	15	72	87	2005	Venezuela	*L. vannamei*	G3, G4
16	44	30	16	2005	Belize	*L. vannamei*	G4
17	45	31	17	2005	Thailand	*L. vannamei*	G4
18	46	32	18	2005	Venezuela	*L. vannamei*	G3, G4
19	47	33	19	2005	Belize	*L. vannamei*	G4
20	48	34	20	2005	Thailand	*L. vannamei*	G4
21	49	35	21	2005	Venezuela	*L. vannamei*	G3
22	50	36	22	2005	Hawaii	*L. vannamei*	G3, G4
23	51	37	23	2005	Hawaii	*L. vannamei*	G3, G4
24	52	38	24	2005	Venezuela	*L. vannamei*	G2–4
25	53	39	25	2005	Hawaii	*L. vannamei*	G3
26	54	43	26	2005	Thailand	*L. vannamei*	G3
27	55	42	27	2005	Hawaii	*L. vannamei*	G3
28	56	40	28	2005	Thailand	*P. monodon*	G3, G4
29	57	41	29	2005	Thailand	*P. monodon*	G3, G4

**Table 2:** A summary of Taura syndrome virus (TSV)-specific primers. Primers for this study were designed based on the TSV reference genome (NC_003005) to amplify TSV using RNA isolated from DFPE tissue. The World Organization for Animal Health (OIE)-recommended primers to amplify TSV through RT-PCR and RT-qPCR are listed for reference.

Primer Name	Location in TSV Genome	Nucleotide Position in the Genome of TSV Reference Strain	Sequence (5′–3′)	Amplicon Size (bp)
TSV 1-F	VP1	7901	GGCGTAGTGAGTAATGTAGCT	137
TSV 1-R	VP1		AGAGACAGGGGTACGCCATA	
TSV 2-F	VP1	7955	ACGAAAGTCAACGCATATGAGA	123
TSV 2-R	VP1		AGGCACTGCAATTGTGGGAT	
TSV 3-F	VP1	8057	GATCCCACAATTGCAGTGCC	125
TSV 3-R	VP1		AGAGACAGGGGTACGCCATA	
TSV 4-F	VP1	8155	TGACACTCCTGATGCGCATG	122
TSV 4-R	VP1		CTAGACTAACTGGGGCAGCG	
TSV 5-F	VP1	8257	CGCTGCCCCAGTTAGTCTAG	122
TSV 5-R	VP1		AGGGGAGATATTGCACCAGC	
TSV 6-F	VP1	8359	GCTGGTGCAATATCTCCCCT	150
TSV 6-R	VP1		GGATCGTACACTCGCATCCA	
TSV 7-F	VP1	8414	TCACAGATCATCGACATCTCACA	124
TSV 7-R	VP1		CACAATCTGCCGTGTACCCA	
TSV 8-F	VP1	8518	TGGGTACACGGCAGATTGTG	145
TSV 8-R	VP1		AAGCGTACCTGGTTCAGCAA	
TSV 9-F	VP1	8643	TTGCTGAACCAGGTACGCTT	149
TSV 9-R	VP1		TTCCCCCAAAGACACCTTCG	
TSV 10-F	VP1	8721	CAGTAACGCGTGCTCCAGTA	147
TSV 10-R	VP1		GCAGTCCGGCATAAGCTAGT	
TSV 11-F	VP1	8834	GGTGGAAGGCACAGACTAGC	121
TSV 11-R	VP1		CAAGAGTTGGAGCGCTGGTA	
TSV 12-F	VP1	8935	TACCAGCGCTCCAACTCTTG	150
TSV 12-R	VP1		TCACCAATCGCTGCCATACT	
TSV 13-F	VP1	9031	TGGTATTTCCGAGGAGACGT	137
TSV 13-R	VP1		TCACTGGAGCTTTGGACTCA	
TSV 14-F	VP1	9071	GCAGCGATTGGTGAAGCTAC	127
TSV 14-R	VP1		TGACCACGGTATAGTTACCTGG	
TSV 15-F	VP2	6948	TGCCTGCTAACCCAGTTGAA	55
TSV 15-R	VP2		AGTCCTCCACTGGTTGTTGT	
TSV 16-F	VP2	7117	AGTCCAGGACCAAGCTCTCA	119
TSV 16-R	VP2		CTGTTGCAAGCTGTTCCTGC	
TSV 17-F	VP2	7233	CAGAATTCAATCAGCCACAC	131
TSV 17-R	VP2		TACTCGTACAGTAACCTCGT	
TSV 18-F	RdRp	5194	CAATGGCCATTGGTTCCGTT	111
TSV 18-R	RdRp		TATACAAGGTAGCGGGGGCT	
TSV 19-F	RdRp	5576	GTGGTTGGGCTCTGAGGAAT	117
TSV 19-R	RdRp		GCCGCAAAAATACCCAAGCT	
TSV 20-F	RdRp	6099	AACCATTCTCAGCCTTCCGG	105
TSV 20-R	RdRp		CCCGTTTTCTCGCTGAGCTA	
TSV 21-F	RdRp	6529	AAACAACGCGCATTGCTTCT	101
TSV 21-R	RdRp		GTACCCTGCGTTCCTACACG	
TSV 9195F *	Intergenic region/Open Reading Frame (ORF) 2	9195	TCAATGAGAGCTTGGTCC	231
TSV 9992R *	Intergenic region/ORF 2		AAGTAGACAGCCGCGCTT	
TSV 1004F **	ORF1	1004	TTGGGCACCCGACATT	72
TSV 1075R **	ORF1		GGGAGCTTAAACTGGACACACTGT	

* OIE-recommended primers (OIE, 2014). ** Primers used for RT-qPCR.

**Table 3:** GenBank accessions utilized for a phylogenetic analysis of the TSV VP1 gene.

GenBank Accession Number	Geographical Origin	Year	Species
FJ876481	Belize	2009	*P. vannamei*
FJ876490	Belize	2009	*P. vannamei*
FJ876507	Nicaragua	2009	*P. vannamei*
FJ876516	Nicaragua	2009	*P. vannamei*
FJ876520	Taiwan	2009	*P. vannamei*
FJ876487	Indonesia	2009	*P. vannamei*
FJ876509	China	2009	*P. vannamei*
AY997025	Thailand	2005	*P. vannamei*
GQ359322	Texas, USA	2010	*P. vannamei*
GQ502201	Texas, USA	2010	*P. vannamei*
FJ876492	Texas, USA	2009	*P. vannamei*
FJ876469	Hawaii	2009	*P. vannamei*
AF277675	Hawaii	1994	*P. vannamei*
FJ876468	Hawaii	2009	*P. vannamei*
FJ876466	Ecuador	2009	*P. vannamei*
FJ876461	Ecuador	2009	*P. vannamei*
FJ876513	Ecuador	2009	*P. vannamei*
FJ876512	Ecuador	2009	*P. vannamei*
FJ7876493	Mexico	2009	*P. vannamei*
DQ000302	Eritrea	2006	*P. monodon*
FJ876495	Eritrea	2009	*P. vannamei*
FJ876508	Aruba	2009	*P. vannamei*
FJ876511	Aruba	2009	*P. vannamei*
FJ876503	Venezuela	2009	*P. vannamei*
DQ212790	Venezuela	2005	*P. vannamei*
FJ876502	Venezuela	2009	*P. vannamei*
JQ356858	Saudi Arabia	2012	*P. indicus*
JX094350	Saudi Arabia	2012	*P. indicus*

**Table 4:** A comparison of quantity, quality and Ct (cycle threshold) values of total RNA isolated with the three RNA extraction kits. The table shows RNA concentrations (ng/µL), 260/280 and 260/230 values and the Ct values for TSV and the elongation factor 1-α (*EF-1α*) gene obtained by RT-qPCR. The mean value and standard deviation in each category are shown. An asterisk (*) indicates a statistically significant difference (*p* < 0.05) between the values of a particular category (mean RNA concentration, mean 260/280, mean 260/230, mean Ct for TSV or mean Ct value for EF-1α) obtained with the three RNA extraction kits.

Extraction Kit	Mean RNA Concentration (ng/µL)	Mean 260/280 Ratio	Mean 260/230 Ratio	Mean Ct Value (TSV)	Mean Ct Value (EF-1α)
Norgen Biotek FFPE RNA Purification Kit	254.90 ± 109.5	1.87 ± 0.05	1.49 ± 0.34	24.12 ± 3.4 *	28.25 ± 2.3
Qiagen RNeasy FFPE Kit	249.20 ± 179.5	1.91 ± 0.06	2.00 ± 0.14	22.90 ± 3.3 *	28.49 ± 1.6
Invitrogen PureLink FFPE RNA Isolation Kit	355.30 ± 207.8	1.86 ± 0.05	1.49 ± 0.27	27.18 ± 3.9	28.68 ± 1.7

**Table 5:** A summary of each sample with the corresponding number of total positive amplification results for each segment of the TSV genome and the total number of positive amplification results per sample.

Sample No.	Total Number of Amplicons Amplified from VP1 Gene (out of 14 Amplicons)	Total Number of Amplicons Amplified fromVP2 Gene (out of 3 Amplicons)	Total Number of Amplicons Amplified from RdRp Gene (out of 4 Amplicons)	Total Number Amplified (of 21 Amplicons)
1	8	3	3	14
2	6	3	3	12
3	0	2	1	3
4	14	3	4	21
5	11	3	3	17
6	12	3	3	18
7	0	2	0	2
8	9	3	3	15
9	3	3	1	7
10	14	3	4	21
11	6	3	3	12
12	7	3	2	12
13	3	3	3	8
14	14	3	4	21
15	13	3	3	19
16	10	3	4	17
17	8	3	0	11
18	12	3	3	18
19	3	3	3	9
20	6	3	4	13
21	12	3	3	18
22	7	3	3	13
23	4	3	0	7
24	4	3	1	8
26	5	3	2	10
27	14	3	4	21
28	0	2	0	2
29	3	3	1	7

## Data Availability

All data can be made available by the corresponding author upon request.

## Supplementary Materials

The following are available online at https://www.mdpi.com/1999-4915/12/9/1030/s1, Figure S1: Agilent TapeStation automated electrophoresis of three representative RNA samples extracted with the Qiagen RNeasy FFPE Kit, Table S1: A summary of primer pairs used to amplify different TSV genes, amplicon size and the total number of samples that displayed positive amplification.

## References

[1] Hasson K.W., Lightner D.V., Poulos B.T., Redman R.M., White B.L., Brock J.A., Bonami J.R. (1995). Taura syndrome in Penaeus vannamei: Demonstration of a viral etiology. Dis. Aquat. Organ..

[2] Lightner D.V., Redman R.M., Pantoja C.R., Tang K.F.J., Noble B.L., Schofield P., Mohney L.L., Nunan L.M., Navarro S.A. (2012). Historic emergence, impact and current status of shrimp pathogens in the Americas. J. Invertebr. Pathol..

[3] Dhar A.K., Cowley J.A., Hasson K.W., Walker P.J. (2004). Genomic Organization, Biology, and Diagnosis of Taura Syndrome Virus and Yellowhead Virus of Penaeid Shrimp. Adv. Virus Res..

[4] Wertheim J.O., Tang K.F.J., Navarro S.A., Lightner D.V. (2009). A quick fuse and the emergence of Taura syndrome virus. Virology.

[5] Valles S.M., Chen Y., Firth A.E., Guérin D.M.A., Hashimoto Y., Herrero S., de Miranda J.R., Ryabov E. (2017). ICTV virus taxonomy profile: Dicistroviridae. J. Gen. Virol..

[6] Bodewes R., van Run P.R.W.A., Schürch A.C., Koopmans M.P.G., Osterhaus A.D.M.E., Baumgärtner W., Kuiken T., Smits S.L. (2015). Virus characterization and discovery in formalin-fixed paraffin-embedded tissues. J. Virol. Methods.

[7] Kenneth W., Hasson J., Aubert H., Redman R.M., Lightner D.V. (1997). A new RNA-friendly fixative for the preservation of penaeid shrimp samples for virological detection using cDNA genomic probes. J. Virol. Methods1997.

[8] Andrade T.P.D., Redman R.M., Lightner D.V. (2008). Evaluation of the preservation of shrimp samples with Davidson’s AFA fixative for infectious myonecrosis virus (IMNV) in situ hybridization. Aquaculture.

[9] Tumpey T.M. (2005). Characterization of the Reconstructed 1918 Spanish Influenza Pandemic Virus. Science.

[10] Wilkins A., Chauhan R., Rust A., Pearson A., Daley F., Manodoro F., Fenwick K., Bliss J., Yarnold J., Somaiah N. (2018). FFPE breast tumour blocks provide reliable sources of both germline and malignant DNA for investigation of genetic determinants of individual tumour responses to treatment. Breast Cancer Res. Treat..

[11] Cruz-Flores R., Mai H.N., Kanrar S., Aranguren Caro L.F., Dhar A.K. (2020). Genome reconstruction of white spot syndrome virus (WSSV) from archival Davidson’s-fixed paraffin embedded shrimp (*Penaeus vannamei*) tissue. Sci. Rep..

[12] Lightner D.V. (1996). A Handbook of Shrimp Pathology and Diagnostic Procedures for Diseases of Cultured Penaeid Shrimp.

[13] Tang K.F.J., Wang J., Lightner D.V. (2004). Quantitation of Taura syndrome virus by real-time RT-PCR with a TaqMan assay. J. Virol. Methods.

[14] Dhar A.K., Kaizer K.N., Lakshman D.K. (2010). Transcriptional analysis of *Penaeus stylirostris* densovirus genes. Virology.

[15] Tamura K., Nei M. (1993). Estimation of the Number of Nucleotide Substitutions in the Control Region of Mitochondrial DNA in Humans and chimpanzees. Mol. Biol. Evol..

[16] Thermo Scientific (2012). NanoDrop Lite: Interpretation of Nucleic Acid 260/280 Ratios.

[17] (2018). Oie Infection with Taura Syndrome Virus. Manual of Diagnostic Tests for Aquatic Animals.

[18] Tang K.F.J., Lightner D.V. (2005). Phylogenetic analysis of Taura syndrome virus isolates collected between 1993 and 2004 and virulence comparison between two isolates representing different genetic variants. Virus Res..

[19] Aranguren L.F., Salazar M., Tang K., Caraballo X., Lightner D. (2013). Characterization of a new strain of Taura syndrome virus (TSV) from Colombian shrimp farms and the implication in the selection of TSV resistant lines. J. Invertebr. Pathol..

[20] Dhar A.K., Lakshman D.K., Amundsen K., Robles-Sikisaka R., Kaizer K.N., Roy S., Hasson K.W., Thomas-Allnutt F.C. (2010). Characterization of a Taura syndrome virus isolate originating from the 2004 Texas epizootic in cultured shrimp. Arch. Virol..

[21] Tang K.F.J., Navarro S.A., Pantoja C.R., Aranguren F.L., Lightner D.V. (2012). New genotypes of white spot syndrome virus (WSSV) and Taura syndrome virus (TSV) from the Kingdom of Saudi Arabia. Dis. Aquat. Organ..

[22] Dwivedi B., Gadagkar S.R. (2009). The impact of sequence parameter values on phylogenetic accuracy. Biol. Med..

