# Supplementary material for: Detection and Phylogenetic Analyses of Taura Syndrome Virus from Archived Davidson’s-Fixed Paraffin-Embedded Shrimp Tissue
Source: Viruses. 2020 Sep 16;12(9):1030. doi: 10.3390/v12091030 (PMC7551232; doi:10.3390/v12091030)
Supplement: Supplementary file 1 [file viruses-12-01030-s001.pdf]

viruses

Supplementary

Detection and Phylogenetic Analyses of Taura Syndrome Virus from Archived Davidson's-Fixed Paraffin-Embedded Shrimp Tissue

Lauren Marie Ochoa, Roberto Cruz-Flores and Arun K. Dhar *

Aquaculture Pathology Laboratory, School of Animal and Comparative Biomedical Sciences, University of Arizona, Tucson, AZ 85721, USA; lauren33@email.arizona.edu (L.M.O.); robertocruz@arizona.edu (R.C.-F.)
* Correspondence: adhar@email.arizona.edu; Tel.: +01-520-621-8727

Abstract: Taura syndrome is a World Organization for Animal Health (OIE)-listed disease of marine shrimp that is caused by Taura syndrome virus (TSV), a single-stranded RNA virus. Here we demonstrate the utility of using 15-year-old archived Davidson's-fixed paraffin-embedded (DFPE) shrimp tissues for TSV detection and phylogenetic analyses. Total RNA was isolated from known TSV-infected DFPE tissues using three commercially available kits and the purity and ability to detect TSV in the isolated RNA were compared. TSV was successfully detected through RT-qPCR in all the tested samples. Among the TSV-specific primers screened through RT-PCR, primer pair TSV-20 for the RNA-dependent RNA polymerase (RdRp), primers TSV-15 and TSV-16 for the capsid protein gene VP2 and primers TSV-5 for the capsid protein gene VP1 amplified the highest number of samples. To assess the phylogenetic relation among different TSV isolates, the VP1 gene was amplified and sequenced in overlapping segments. Concatenated sequences from smaller fragments were taken for phylogenetic analyses. The results showed that the TSV isolates from this study generally clustered with homologous isolates from the corresponding geographical regions indicating RNA derived from DFPE tissues can be used for pathogen detection and retrospective analyses. The ability to perform genomic characterization from archived tissue will expedite pathogen discovery, development of diagnostic tools and prevent disease spread in shrimp and potentially other aquaculture species worldwide.

Keywords: Taura syndrome virus; aquaculture; shrimp

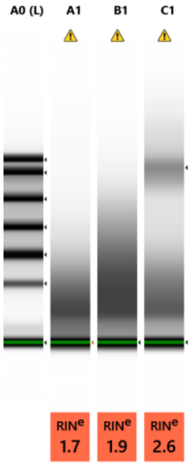

Figure S1. Agilent TapeStation automated electrophoresis of three representative RNA samples extracted with the Qiagen RNeasy FFPE Kit. All three samples presented very low RNA Integrity Numbers (RIN) that ranged from 1.7–2.6, indicating a high level of degradation and fragmentation. The tested samples were derived from Case 2 (A1), Case 13 (B1) and Case 23 (C1). The RNA concentration for these samples was 43.6 ng/µL, 73.5 ng/µL and 56.5 ng/µL for cases 2 (A1), 13 (B1) and 25 (C1) respectively.

Table S1. A summary of primer pairs used to amplify different TSV genes, amplicon size and the total number of samples that displayed positive amplification.

Primer Set used for the amplification	Target gene amplified	Total number of positive amplification (out of 28)
EF-1α	EF-1α	28
TSV-1	VP1	20
TSV-2	VP1	13
TSV-3	VP1	15
TSV-4	VP1	15
TSV-5	VP1	25
TSV-6	VP1	15
TSV-7	VP1	6
TSV-8	VP1	10
TSV-9	VP1	12
TSV-10	VP1	9
TSV-11	VP1	18
TSV-12	VP1	16
TSV-13	VP1	16
TSV-14	VP1	17
TSV-15	VP2	28
TSV-16	VP2	28
TSV-17	VP2	25
TSV-18	RdRp	16
TSV-19	RdRp	11
TSV-20	RdRp	20
TSV-21	RdRp	22
OIE Recommended Primers	TSV ORF1/2 Intergenic region	03
